# Effects of Cannabidiol Oil on Anesthetic Requirements in Cats: MAC Determination and Serum Profiling via Nanoscale Liquid Chromatography–Tandem Mass Spectrometry

**DOI:** 10.3390/ani15101393

**Published:** 2025-05-12

**Authors:** Panisara Suriyawongpongsa, Sirirat Niyom, Kannika Wanapinit, Monchanok Vijarnsorn, Sittiruk Roytrakul, Sekkarin Ploypetch

**Affiliations:** 1 Veterinary Clinical Studies Program, Faculty of Veterinary Medicine, Graduated School, Kasetsart University, Nakorn Pathom 73140, Thailand; panisara.sur@ku.th; 2 Department of Companion Animal Clinical Sciences, Faculty of Veterinary Medicine, Kasetsart University, Bangkok 10900, Thailand; monchanok.vi@ku.th; 3 Kasetsart University Veterinary Teaching Hospital, Faculty of Veterinary Medicine, Kasetsart University, Bangkok 10900, Thailand; kannika.wana@ku.th; 4 National Center for Genetic Engineering and Biotechnology, National Science and Technology Development Agency, Pathum Thani 12120, Thailand; sittiruk@biotec.or.th; 5 Department of Clinical Sciences and Public Health, Faculty of Veterinary Science, Mahidol University, Nakhon Pathom 73170, Thailand; sekkarin.plo@mahidol.ac.th

**Keywords:** anesthetic requirement, cannabidiol, cat, isoflurane, minimum alveolar concentration, proteomic analysis

## Abstract

Cannabidiol (CBD), a non-psychotropic cannabinoid derived from *Cannabis* plants, is increasingly explored for its potential therapeutic applications in veterinary medicine. This study aimed to evaluate the impact of CBD oil on the minimum alveolar concentration of isoflurane (MAC_iso_) in cats. Sixteen healthy cats underwent isoflurane anesthesia, and the MAC_iso_ was determined using the tail-clamping technique both at baseline and 30 min after the administration of CBD oil (2 mg/kg) via a stomach tube. CBD administration resulted in a significant 11% reduction in the MAC_iso_, from 1.77 ± 0.14% to 1.62 ± 0.21% (*p* < 0.001). Following CBD administration, heart and respiratory rates were elevated at the time of MAC_iso_ determination compared to baseline whereas other physiological parameters remained unchanged. Serum biochemical analysis conducted two weeks post administration revealed a significant decrease in blood urea nitrogen (BUN) levels while one cat exhibited a mild increase in alanine aminotransferase (ALT). Proteomic analysis identified 12 CBD-associated proteins in feline serum 30 min post administration, with CBDA and THCA synthases demonstrating significant upregulation. These findings indicate that CBD oil reduces anesthetic requirements in cats without inducing significant physiological disturbances. Further research is warranted to elucidate the underlying mechanisms of CBD’s anesthetic-sparing effects and its implications in veterinary anesthesia.

## Simple Summary

Cannabinoids are increasingly studied in veterinary medicine for their potential therapeutic benefits in companion animals; however, scientific evidence remains limited. While cannabidiol (CBD) has been shown to reduce seizures, relieve stress, alleviate osteoarthritis (OA) pain, and improve the quality of life in dogs with OA, its effects in cats are less understood. This study evaluated the impact of CBD oil on anesthesia in cats by measuring the minimum alveolar concentration (MAC) of isoflurane before and after administering 2 mg/kg of CBD to sixteen healthy cats. The results showed a significant reduction in the MAC following CBD administration, indicating decreased anesthetic requirements. Physiological parameters during anesthesia and blood analyses revealed no severe adverse effects. Heart and respiratory rates were slightly elevated after CBD administration. Blood urea nitrogen (BUN) levels significantly decreased two weeks post administration while one cat exhibited a mild increase in alanine aminotransferase (ALT). These findings suggest that CBD oil may reduce anesthetic requirements and is well tolerated in healthy adult cats. However, further studies are needed to explore the mechanisms underlying CBD’s anesthetic-sparing effects and its broader clinical applications in feline anesthesia.

## 1. Introduction

Cannabidiol (CBD) is a non-psychotropic cannabinoid derived from *Cannabis* plants, distinct from delta-9-tetrahydrocannzaabinol (THC), which exhibits pronounced psychoactive effects [1]. In human medicine, CBD has been investigated for its potential therapeutic applications, including for epilepsy, neurodegenerative diseases, anxiety, inflammation, and pain management [2]. Over the past few decades, interest in cannabinoids has grown in veterinary medicine, driven by the rapid expansion of the hemp industry and the increasing legalization of marijuana, which have broadened the commercial availability of cannabinoid products [3]. Consequently, the exploration of cannabinoid applications in animal health and therapeutics has gained momentum.

Despite the increasing accessibility of cannabinoid products, scientific evidence regarding their efficacy and safety in animals remains limited. Research on cannabinoids in companion animals is still in its early stages. Some studies in dogs have suggested that CBD may reduce seizure frequency in idiopathic epilepsy [4], alleviate pain and improve the quality of life in osteoarthritic (OA) patients [5,6,7], mitigate stress associated with caregiver separation [8], and have anti-inflammatory and immuno-modulating properties [9]. Pain alleviation has also been observed in horses with OA following the oral administration of cannabidiolic acid (CBDA), a combination of cannabigerol and cannabidiol (CBG/CBD) oil, or CBD in conjunction with phenylbutazone [10,11]. In contrast, CBD research in cats is even more limited, focusing primarily on appropriate dosages and potential adverse effects [12,13,14], while evidence supporting its therapeutic benefits remains scarce [15]. These knowledge gaps highlight the need for further investigation into the clinical effectiveness of CBD in this species.

Studies have documented several adverse effects of CBD in humans and laboratory animals, including the inhibition of hepatic drug metabolism, alterations in in vitro cell viability, reduced fertilization capacity, and the suppression of p-glycoprotein and other drug transporters [16]. Meanwhile, research across various CBD concentrations has found no significant effects on physiological parameters such as the heart rate, blood pressure, and body temperature in humans, mice, rats, and horses [10,16]. In cats, CBD administration has been associated with lethargy, hypersalivation, and increased liver enzyme levels [12,13]. Similarly, prolonged CBD administration in dogs has been linked to elevated alkaline phosphatase levels [6].

Given its potential therapeutic analgesic and anxiolytic properties [5,6,7,8,10,11] and minimal impact on physiological parameters [10,16], CBD may serve as an adjunct in veterinary anesthesia. Since most anesthetic agents induce dose-dependent cardiopulmonary depression, strategies to reduce anesthetic requirements are of clinical interest. Conventional tranquilizers and sedatives, such as phenothiazines and alpha-2 adrenergic agonists, effectively lower anesthetic needs but are often associated with significant cardiovascular effects [17]. In contrast, CBD appears to exert fewer physiological disturbances, suggesting its potential as a safer alternative. Furthermore, its multimodal effects on the endocannabinoid system, including the modulation of pain, inflammation, and anxiety [18], indicate that it may complement existing anesthetic protocols.

This study investigated the medicinal potential of CBD in veterinary anesthesia, focusing on its effects on anesthetic requirements as determined by the minimum alveolar concentration of isoflurane (MAC_iso)_ using tail-clamp noxious stimulation in cats. Additionally, it included the development and validation of a nanoscale liquid chromatography–tandem mass spectrometry (nanoLC-MS/MS) method for the precise detection and quantification of CBD in feline serum. The accurate measurement of cannabinoids is essential not only for pharmacokinetic assessment but also for monitoring animals exposed to cannabis-derived compounds.

## 2. Materials and Methods

### 2.1. Animals

The study was approved by the Kasetsart University Animal Care and Use Committee (Approval ID: ACKU64-VET-078). Sixteen healthy cats that comprised ten domestic shorthair (DSH), five Persians, and one British shorthair were enrolled in the study. The average body weight was 4.61 ± 0.84 kg, and the mean age was 6.56 ± 2.5 years. The group consisted of six castrated males, nine spayed females, and one intact female. Written consent for participation was obtained from the responsible owner. Inclusion criteria required cats to be between 1 and 10 years old, clinically healthy, with no diagnosed conditions or medication use within one month. All cats underwent a physical examination, complete blood count, and blood chemistry analysis to confirm normal health status before enrollment. Cats with abnormal blood profiles or physical examination findings were excluded. The sample size was calculated based on preliminary data from two cats measuring MAC_iso_ before and after CBD administration. The mean difference in MAC was estimated at 0.15, with a standard deviation of the difference of 0.21. Using these parameters (alpha = 0.05, power = 0.80), a sample size of 16 cats was determined to be sufficient to detect a statistically significant effect.

### 2.2. Experimental Design

This study was conducted in two sequential phases: a preliminary phase, designed to determine the time frame in which cats begin to exhibit effects, followed by an experimental phase aimed at evaluating the main objectives. The two phases were conducted approximately one month apart. Cats enrolled in the preliminary phase did not undergo MAC value assessment until after a four-week washout period to eliminate potential carryover effects. The tested CBD product was a pure CBD isolate with a purity of 99.52% CBD and delta-8 and delta-9 THC levels below 0.005% (PURE CBD Isolate; Pure Production AG, Zeiningen, Switzerland). The formulation contained 40 mg of CBD per 1 mL.

In the preliminary phase, five healthy cats (two females and three males), aged between 2 and 10 years and weighing 3.6 to 5.5 kg, were enrolled. The group consisted of two Persian cats and three DSH cats. All cats were fasted overnight, and their access to water was restricted two hours prior to administration, in accordance with the protocol used in the experimental phase. CBD oil was administered at a dose of 2 mg/kg via direct oral administration, and the cats’ behavior was monitored to estimate the onset and duration of sedative effects using a sedation score. A numerical sedation scoring system adapted from previous feline studies [19,20,21,22] was used in this study (Table A1). Minor modifications were made to enhance practicality under the current experimental conditions. In particular, a moving toy was introduced as an additional stimulus alongside gentle stroking and handclapping to assess the animal’s responsiveness. The sedation scoring system was applied to assess behavior before CBD administration and at 15, 30, 45, and 60 min post administration. The evaluation was performed by a single investigator for all cats at each time point.

During the experimental phase, sixteen cats were included. Food was withheld for 12 h while water was restricted for 2 h prior to anesthesia with isoflurane (Piramal Critical Care, Inc., Bethlehem, USA) in oxygen. Preoxygenation was performed for 5 min in an anesthetic chamber before anesthesia induction. Anesthesia was induced in the chamber using an oxygen flow rate of 10 L/min with a 5% isoflurane vaporizer setting. Once anesthetized, the cats were removed from the chamber, and a face mask was used to deepen anesthesia to allow endotracheal intubation. Intubation was performed using an appropriately sized endotracheal tube (internal diameter: 3.5–4.5 mm) when jaw tone was lost. After intubation, the cats were then connected to a Bain circuit and maintained on isoflurane (2% at 2 L/min oxygen).

To continuously collect respiratory gas samples, a 5 French (5F/OD 1.65 mm × 50 cm) feeding tube was inserted inside the endotracheal tube, with its tip positioned at the distal end of the endotracheal tube or at the level of the tracheal carina. The feeding tube was then connected to a gas analyzer (Dräger Vamos Plus infrared gas analyzer, Dräger Medical, Lübeck, Germany). The cats were allowed to breathe spontaneously throughout the procedure. Inspired oxygen concentration was maintained above 90%, with a minimum peripheral arterial oxygen saturation (SpO_2_) of 96% and an end-tidal carbon dioxide partial pressure (ETCO_2_) between 35 and 45 mmHg (4.7–6 kPa). If ETCO_2_ exceeded 45 mmHg, mechanical ventilation was initiated.

A 22-to-24-gauge catheter (Becton Dickinson Infusion Therapy System Inc., Sandy, USA) was placed percutaneously into the right cephalic vein for fluid administration and into the left cephalic vein for blood sampling. Acetate Ringer’s solution (General Hospital Products Public Co., Ltd., Pathum Thani, Thailand) was infused at a rate of 3 mL/kg/h using an infusion pump throughout anesthesia. The cats were positioned in dorsal recumbency. A circulating warm-water blanket (Soarmed Medical-Tech Co., Ltd., Taipei City, Taiwan) and a forced warm air blanket (COCOON Convective Warming System CWS 4000; Care Essential Pty., Ltd., North Geelong, Australia) were used to maintain core body temperature between 37 and 39 °C.

Physiological parameters were continuously monitored using a multi-parameter patient monitor (Datex-Ohmeda CARESCAPE Multifunctional Anesthesia Monitor; GE Healthcare, Helsinki, Finland). These parameters included heart rate (HR), respiratory rate (RR), electrocardiography (ECG), systolic arterial blood pressure (SAP), mean arterial blood pressure (MAP), diastolic arterial blood pressure (DAP), SpO_2_, ETCO_2_, end-tidal isoflurane concentration, and body temperature. HR and cardiac rhythm were assessed using lead II ECG. Non-invasive oscillometric blood pressure measurements were performed to monitor SAP, MAP, and DAP. RR, ETCO_2_, and end-tidal isoflurane concentration were measured with a Dräger Vamos Plus infrared gas analyzer (Dräger Medical, Lübeck, Germany). The gas analyzer was calibrated before each experiment using a standardized calibration gas mixture (1% isoflurane and 1% sevoflurane in 5% CO_2_ and 70% N_2_O; Air Liquide Healthcare America Co., Radnor, USA). A temperature probe was inserted rectally into the distal colon for continuous temperature monitoring.

#### 2.2.1. MAC Determination Method

Approximately 60 min was spent on instrumentation after intubation. The isoflurane vaporizer was then adjusted to achieve a constant end-tidal isoflurane concentration of 2% and was maintained for at least 15 min to allow for equilibration before initiating the determination of the baseline minimum alveolar concentration of isoflurane (MAC_baseline_) using the tail clamp technique. In brief, a large Rochester-Carmalt forceps, with each jaw covered by a soft protective plastic tube to prevent tail injury, was applied for a maximum of 60 s or until each cat exhibited a positive purposeful response. Tail clamping was initiated at a randomly selected site approximately 5–10 cm from the base of the tail [23,24] to minimize tissue damage. The forceps were consistently clamped close up to the first ratchet lock but not fully closed [23]. Clamping was immediately discontinued if a gross purposeful movement was observed before completing the 60 s duration. A positive response was defined as a gross purposeful movement in response to the noxious stimulus, such as jerking or twisting of the head, running, or clawing of the limbs. Other signs, such as an increased respiratory rate, swallowing, or coughing, were not considered positive responses. If a positive response was observed, the end-tidal isoflurane concentration was increased by 10–20%, followed by a minimum 15 min anesthetic re-equilibration period before reassessment. If no purposeful movement occurred within 60 s (negative response), tail clamping was discontinued, and the end-tidal isoflurane concentration was reduced by 10–20% for the next determination. The mean of a positive and negative response was regarded as a single MAC value. Tail clamping for noxious stimulation was consistently performed by the same operator throughout the study to minimize variability in the stimulus application.

The MAC_baseline_ was measured in triplicate, and the final value was calculated as the average of the three MAC values. This MAC_baseline_ was determined before administering CBD oil and served as a reference for calculating the percentage of increase or reduction. Each adjustment of the end-tidal isoflurane concentration was followed by a minimum 15 min equilibration period before the next measurement.

After determining MAC_baseline_, a 10 French (10F/OD 3.3 mm × 50 cm) feeding tube was inserted through the oropharynx, passed through the esophagus, and positioned in the stomach. The tube’s placement was confirmed using C-arm fluoroscopy (Siemens AG, Wittelsbacherplatz 2, DE 80333, Munich, Germany). A CBD dosage of 2 mg/kg was then administered, followed by 2–3 mL of sterile water to flush any remaining CBD oil from the tube. Once the CBD oil had completely entered the stomach, the end-tidal isoflurane concentration was adjusted to the cat’s MAC_baseline_. The MAC_iso_ after CBD administration (MAC_CBD_) was assessed in triplicate, with measurements beginning at least 15 min post administration to allow for equilibration and coinciding with the sedation effect observed between 15 and 45 min post CBD in the preliminary phase. The final MAC_CBD_ value was calculated as the average of three MAC values measured after CBD administration.

Physiological parameters, including HR, RR, SAP, DAP, MAP, SpO_2_, ETCO_2_, and body temperature, were continuously monitored during anesthesia. All parameters were measured and recorded about 30 s before stimulation. For each MAC point, values were determined by averaging the measurements taken immediately before stimuli that resulted in either a positive or negative response, both at baseline and following CBD administration for further analysis. Upon completion of the experimental protocol, meloxicam (0.3 mg/kg) was administered subcutaneously to all cats. Owners were requested to bring their cats for a follow-up physical examination, hematology, and blood chemistry assessment 1–2 weeks after the study to evaluate their health status.

Blood samples (2 mL) were collected before MAC_baseline_ determination and 30 min after CBD administration. Serum was separated by centrifuging the blood samples in plain tubes at 4000 rpm for 5 min. After centrifugation, the serum was stored at −80 °C to preserve sample quality and stability until further analysis [25]. Total protein concentrations in the serum were measured using Lowry’s assay, with bovine serum albumin as the standard [26].

#### 2.2.2. Statistical Analysis

Descriptive and statistical analyses were conducted using R software (version 4.2.1; R Core Team, 2022, Vienna, Austria). The distribution of each variable was evaluated using the Shapiro–Wilk normality test. Data following a normal distribution were presented as means ± standard deviations (SDs) while non-normally distributed variables were expressed as medians and interquartile ranges (IQRs). A paired *t*-test was used for parametric comparisons between pre-treatment and post-treatment values whereas the Wilcoxon signed-rank test was applied for non-parametric data. A *p*-value < 0.05 was considered statistically significant.

### 2.3. Analysis of Serum Protein by In-Solution Digestion Coupled with nanoLC-MS/MS

#### 2.3.1. In Solution Digestion

Protein samples were prepared by reducing disulfide bonds with 5 mM dithiothreitol in 10 mM ammonium bicarbonate (NH_4_HCO_3_) at 60 °C for 1 h. Reduced cysteine residues were then alkylated by incubation with 15 mM iodoacetamide in 10 mM NH_4_HCO_3_ at 25 °C for 45 min. Following this, proteins were digested with trypsin (Promega, Madison, WI, USA) at room temperature for 3 h. The resulting peptide samples were dissolved in 0.1% formic acid (FA) and prepared for LC-MS/MS analysis.

#### 2.3.2. NanoLC-MS/MS

Peptide identification was performed using the Ultimate 3000 Nano/Capillary LC System (Thermo Scientific, Waltham, MA, USA) coupled with a ZenoTOF 7600 mass spectrometer (SCIEX, Framingham, MA, USA). Digested peptide samples were first concentrated using Acclaim™ 5 µm PepMap™ 300 µ-Precolumns™ packed with C18 material (300 µm × 5 mm, 5 µm, 100 Å, Thermo Scientific) and then separated using an Acclaim™ PepMap™ RSLC column (75 µm × 15 cm, 2 µm, 100 Å, Thermo Scientific) maintained at 35 °C. Protein separation was carried out using solvents A (0.1% formic acid in water) and B (0.1% formic acid in 80% acetonitrile) with a gradient of solvent B from 5% to 55% over 30 min at a flow rate of 0.30 µL/min. The ZenoTOF 7600 system’s source parameters were configured as follows: ion source gas 1 at 8 psi, curtain gas at 35 psi, collision-activated dissociation (CAD) gas at 7 psi, source temperature at 200 °C, spray voltage at 3300 V, and positive polarity mode. For data-dependent acquisition (DDA), the top 50 most abundant precursor ions from each MS1 survey were selected for MS/MS analysis, provided their intensity exceeded 150 cps. Dynamic exclusion was applied for 12 s after two MS/MS samplings per precursor ion. MS2 spectra were collected over a mass range of 100–1800 *m/z* with a 50 ms accumulation time. Collision energy settings included an 80 V declustering potential, no declustering potential (DP) spread, and a capillary electrophoresis (CE) spread of 0 V. The Zeno trap threshold was set to 150,000 cps, and time bins were summed across all channels. The Top 60 DDA method operated with a 3.0 s cycle time. Quality control was ensured by analyzing three replicates of the same sample to evaluate reproducibility. Additionally, bovine serum albumin digestion was used as a control to monitor the performance and reliability of the mass spectrometer and overall analytical workflow, following previously established protocols [27,28].

#### 2.3.3. Serum Proteome Processing and Analysis

For protein identification, raw mass spectral data were analyzed using MaxQuant software (version 2.2.0.0) to identify peptides and proteins. MS/MS data were searched against the reference proteome database for the target *Cannabis sativa* (hemp) (marijuana), obtained from UniProt, supplemented with a contaminant database. Protein identification was considered significant with a *p*-value < 0.05 and a false discovery rate (FDR) of 1% applied to both peptides and proteins. Peptides were required to have a minimum length of 7 amino acid residues and include at least one unique peptide, as described in prior studies [29,30]. Label-free quantification was performed using the MaxLFQ algorithm in MaxQuant, with a minimum ratio count of 2. The abundance of 46 CBD-associated proteins in cat serum was analyzed by targeting the organism of *Cannabis sativa* (hemp) (marijuana) proteins (Supplement Table S1), identified from reviewed entries in the UniProt Swiss-Prot database (1 January 2025) [31]. CBD-associated proteins in cat plasma were searched by targeting “tetrahydrocannabinolic acid (THCA) synthase and cannabidiolic acid (CBDA) synthase” from the protein list. Protein abundances were compared across all time points using MetaboAnalyst (version 6.0) (www.metaboanalyst.ca) [32]. Statistical significance between groups was assessed using fold change (FC) = 2.0 and the Wilcoxon signed-rank test, with a threshold of *p* < 0.05.

## 3. Results

### 3.1. Preliminary Phase

All five cats prior to receiving CBD oil orally at a dose of 2 mg/kg were fully awake, demonstrating normal standing and walking abilities (sedation score = 0). At 15 min post administration, four cats remained standing and walking but exhibited signs of staggering when attempting to walk (sedation score = 1). One cat displayed sternal recumbency and weakness in standing or rising (sedation score = 2). By 30 min post administration, all cats exhibited sternal recumbency, were able to lift their heads, but showed weakness in standing or attempting to rise (all sedation scores = 2). At 45 min, two cats initiated attempts to walk while three cats remained in sternal recumbency. By 60 min post administration, most cats returned to their usual behaviors, including walking steadily, grooming, and demonstrating environmental awareness. A Friedman test was conducted to evaluate differences in scores across time points. The results revealed a statistically significant effect of time, χ^2^(4) = 18.89, *p* = 0.00082, indicating that the scores changed significantly over the different time intervals.

### 3.2. MAC Determination

The mean total anesthesia duration was 202.19 ± 34.15 min. The median induction time was 10 (6.8–10) min. The time spent determining MAC_baseline_ and MAC_CBD_ was 45.5 (41.5–57.3) and 43 (36.5–69) min, respectively, with no significant difference between the two determinations (*p* = 0.896). The median time from isoflurane discontinuation to extubation was 5 (5–6.3) min. All cats fully recovered and were discharged without complications at the end of the study.

The MAC values at the baseline and post CBD administration, along with corresponding physiological parameters, are presented in Table 1. MAC_CBD_ was significantly lower than MAC_baseline_ (*p* < 0.001), representing an 11% reduction. Body temperature, SAP, MAP, DAP, ETCO_2_, and SpO_2_ did not differ significantly between the baseline and post CBD administration. No arrhythmias were observed in any cat during the experiment. However, the HR and RR increased significantly compared to baseline (*p* < 0.001 and *p* = 0.005, respectively).

The hematology and blood chemistry profiles of the cats before treatment (baseline) and 1–2 weeks after receiving 2 mg/kg CBD are presented in Table 2. Blood urea nitrogen (BUN) was the only parameter that changed significantly (*p* = 0.010). However, serum alanine aminotransferase (ALT) in one cat increased from 46 U/L at the baseline to 143 U/L, exceeding the reference interval [33].

### 3.3. Proteomic Analysis in Cat’s Serum for the Target Cannabis Sativa

**Figure 1:**
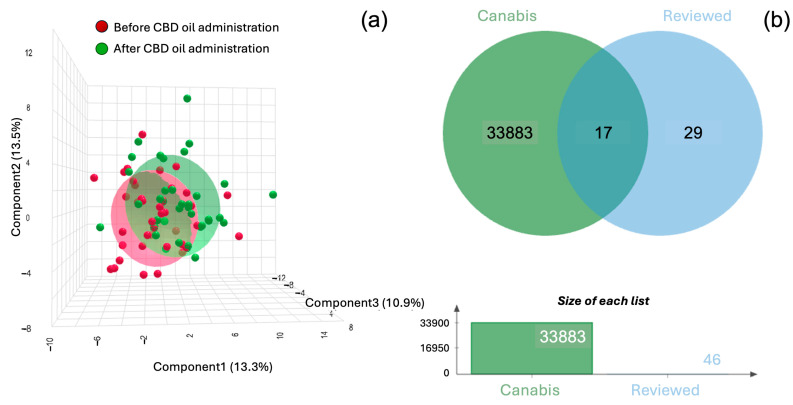
A 3D scatter plot (**a**) depicts the spatial distribution of data from two groups of cats: red dots represent samples collected before CBD oil administration while green dots represent samples collected after administration. Overlapping regions between the two groups suggest shared characteristics or similar proteomic profiles. A Venn diagram (**b**) compares two datasets: 33,900 proteins labeled “Cannabis” (green) and 46 CBD-associated proteins identified from reviewed entries in the UniProt Swiss-Prot database as of 1 January 2025 (blue). Seventeen proteins were found to be common between the two datasets. A bar chart below the Venn diagram illustrates the relative sizes of each dataset.

**Figure 2:**
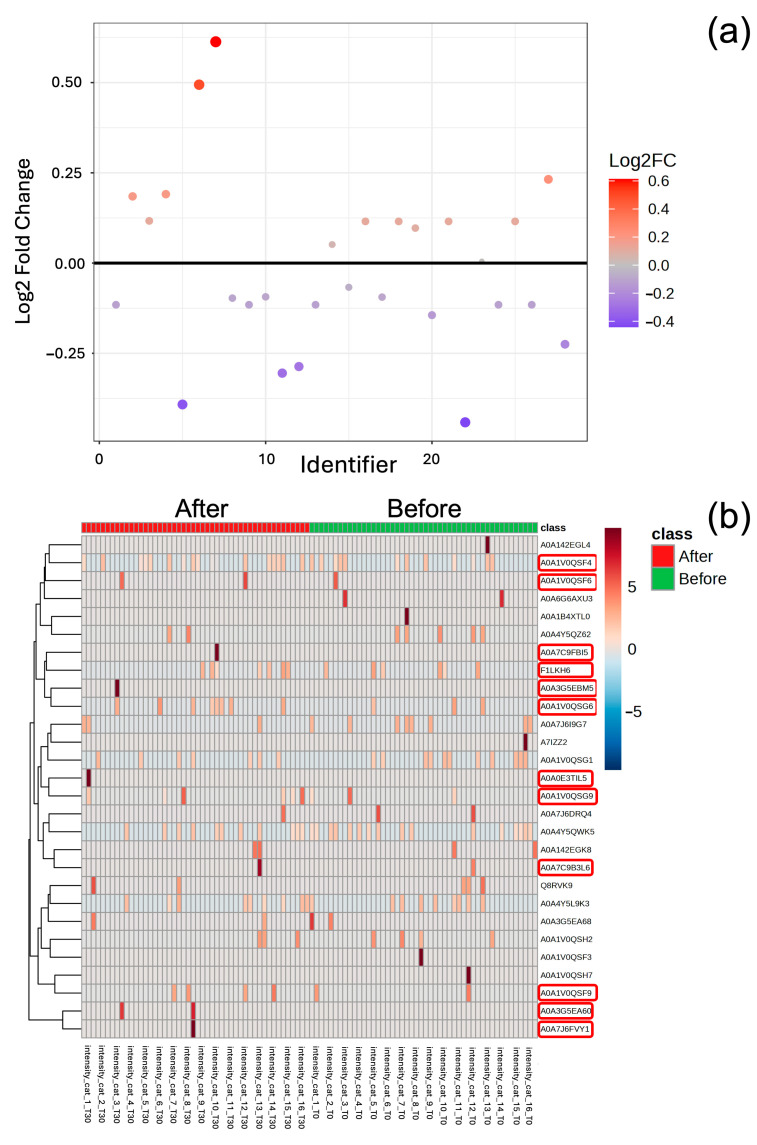
Proteins with a Log2 Fold Change (FC) greater than 1.0 are considered upregulated (red dots) while those with a Log2 FC less than −1.0 are downregulated (blue dots). The analysis revealed 12 upregulated and 15 downregulated proteins at FC 1.0 (**a**). Heatmap illustrating the expression levels of proteins before and after treatment. Red indicates upregulation while blue indicates downregulation. Twelve proteins (indicated by red circles) showed significant upregulation following treatment (**b**).

A comprehensive analysis identified 33,900 proteins from *Cannabis sativa* (hemp)(marijuana) that were successfully matched (Supplement Table S2). The resulting three-dimensional PLS-DA plot showed noticeable clusters between the results before and after CBD oil administration. A Venn diagram (Figure 1b) was used to visually represent the overlap between CBD-related proteins identified in cat serum and the 46 reviewed entries. Among these, 17 proteins were identified as common CBD-associated proteins in cat serum. The dynamics of these 17 proteins, along with 13 THCA/CBDA synthase proteins, were further analyzed using paired fold change (FC = 2.0) to identify differentially expressed proteins before and after CBD administration (Supplement Table S3). When plotted on a log_2_ scale, the analysis revealed 12 upregulated and 15 downregulated proteins at FC = 1.0 (Figure 2a).

Heatmaps (Figure 2b) were generated to cluster proteins based on expression levels, illustrating clear differences between the results pre and post CBD administration. Notably, significant expression changes were observed, with 12 CBD-related proteins upregulated following oral CBD administration. These proteins were associated with various biological processes, cellular components, and molecular functions (Table 3). Among the THCA/CBDA synthase proteins analyzed, those with gene names—including tetrahydrocannabinolic acid synthase (THCAS) and cannabidiolic acid synthase (CBDAS)—were considered more reliable and credible for functional characterization and comparative analysis.

## 4. Discussion

This study evaluated the effects of CBD oil on anesthetic requirements in cats, specifically its impact on the inhalant anesthetic MAC, and demonstrated that a single dose of 2 mg/kg CBD significantly reduced the MAC_iso_. These findings were consistent with previous studies in various animal species, supporting the anesthetic-sparing effects of cannabinoids. In dogs, the intravenous administration of THC at 0.5 and 2 mg/kg reduced the MAC of halothane [34], while in rats, the gastric administration of 10 mg/kg THC decreased the MAC of sevoflurane [35]. Additionally, in rodents, cannabinoid receptor agonists have been shown to prolong isoflurane-induced sleep in mice [36] while rats pretreated with 20 mg/kg CBD have exhibited a significantly shorter induction time for isoflurane anesthesia [37]. For injectable anesthesia, a propofol-sparing effect has been observed in dogs that have received 6 mg/kg of a full-spectrum CBD-rich extract [38] and in those administered phytocannabinoids intraperitoneally, which have also exhibited a more rapid onset and prolonged duration of anesthesia [39]. The proposed mechanism underlies these anesthetic-sparing effects of cannabinoids such as CBD binding to postsynaptic cannabinoid receptor type 1 (CB1), which subsequently activates gamma-aminobutyric acid type A (GABA_A_) receptors, enhancing their inhibitory effects on neurotransmission and thereby reducing anesthetic requirements [40,41]. Another potential mechanism is the inhibition of fatty acid amide hydrolase (FAAH) by propofol, which increases brain endogenous cannabinoids such as anandamide (AEA), contributing to its sedative and anesthetic properties [42].

In contrast, some studies have reported that THC is associated with a reduction in the sedative and anesthetic effects of anesthetic agents [43,44]. The proposed mechanism involves THC binding to presynaptic CB1 receptors, inhibiting GABA release and thereby reducing inhibitory neurotransmission [40]. Consequently, THC may increase the requirement for GABAergic inhalation and intravenous anesthetics to achieve an adequate depth of anesthesia. Additionally, cannabinoids influence the expression and activity of cytochrome P450 (CYP) enzymes, accelerating the metabolic degradation of propofol [40,44], which may further increase propofol requirements. In humans, chronic cannabis users have been reported to require higher doses of intravenous and inhalant anesthetic agents to achieve adequate sedation or anesthesia [44,45,46,47,48,49]. This effect may be attributed to CB1 receptor downregulation in the brain [50] and lower circulating endocannabinoid levels [51], which have been associated with long-term cannabis use.

These discrepancies among studies may be attributed to differences in the cannabinoid composition (e.g., varying ratios of THC to CBD), administration patterns (single dose vs. chronic exposure), or interspecies variations in cannabinoid pharmacokinetics, receptor distributions, and signaling pathways. Further research is needed to clarify these variations. Nevertheless, based on the findings of this study and the proposed mechanisms of CBD, it is plausible that CBD may be beneficial as an adjunct agent for feline anesthesia.

By reducing isoflurane requirements, CBD oil may help mitigate dose-dependent cardiopulmonary suppression, a critical concern in inhalation anesthesia [52]. In this study, blood pressure (SAP, MAP, and DAP) remained stable throughout the experiment, and no arrhythmias were observed in any cat under anesthesia, even after CBD co-administration. Interestingly, the HR and RR were higher but did not exceed normal limits during MAC_CBD_ determination compared to during MAC_baseline_ determination, possibly indicating reduced cardiopulmonary depression due to the lower isoflurane concentration required after CBD administration.

The 2 mg/kg dose was selected mainly based on prior research demonstrating its anti-anxiety and analgesic efficacy in dogs while maintaining a favorable safety profile [5,6,8]. A low dose was deliberately chosen to minimize potential adverse effects. Prior to the experiment, a preliminary trial was conducted in five healthy cats to assess whether the drug induced sedation or nervous system depression, potentially reducing anesthetic requirements, and to determine the duration of its effects. The onset of sedation was observed at approximately 15 min post administration, with effects beginning to wear off at around 45 min. Based on these findings, MAC determination was performed between 15 and 45 min after CBD administration. However, one of the limitations of this study was the lack of additional time points, such as at 60 min, to assess whether the effect persisted or had completely dissipated. This omission was intentional to minimize discomfort as repeated MAC testing could pose a risk of inducing pain or discomfort upon recovery. Another limitation of this study lay in the small number of animals included in the preliminary phase. Although the data obtained were valuable for estimating the onset of observable effects following CBD administration, the limited sample size restricts the robustness and generalizability of these findings. This limitation was further compounded in the experimental phase, where financial constraints also contributed to a small sample size and the absence of a placebo control group. Both factors limit the broader applicability of the results and necessitate caution in their interpretation. In addition, all MAC determinations were performed by a single investigator who was aware of the treatment protocol. While this helped ensure consistency, it may have introduced observer bias. Future studies with larger sample sizes, the inclusion of placebo controls, and blinded assessments by multiple investigators are warranted to enhance the reliability and objectivity of the findings.

A mild elevation in serum ALT was observed in one cat, with no associated clinical signs of liver dysfunction. This increase may be related to the hepatic metabolism of CBD via cytochrome P450 enzymes, likely reflecting increased hepatic workload rather than significant liver damage [53]. Similar ALT elevations have been reported in dogs and cats receiving comparable or repeated doses of CBD [6,13]. Interestingly, the cat with elevated ALT had had a prior episode of elevated liver enzymes the previous year, which resolved with treatment. At the time of study enrollment, the cat met the inclusion criteria, as it was not under medication, had no clinical signs of disease, and was deemed healthy based on physical examination and normal hematology and biochemistry results. This finding raises concerns about the safety of CBD use not only in animals with active liver disease but also in those with a history of liver conditions.

The observed reduction in BUN levels in cats two weeks after CBD administration may have been influenced by fluid administration during anesthesia, which could have diluted BUN levels. However, it is also possible that CBD contributed to this effect. Some evidence suggests that CBD’s anti-inflammatory properties may help mitigate kidney inflammation, improve renal function, and enhance renal hemodynamics [54]. While these findings are intriguing, there is no concrete evidence from clinical trials demonstrating its direct efficacy in treating or improving kidney function in patients.

This paper provides a comprehensive proteomic analysis of cannabinoids in feline serum, highlighting significant changes in protein expression. Nanoscale LC-MS/MS was employed not only to quantify CBD in feline serum but also to detect key enzymes involved in cannabinoid biosynthesis, specifically CBDA synthase and THCA synthase [55]. These enzymes are associated with the biosynthesis of CBDA and THCA—compounds shown to exert anti-inflammatory, anti-hyperalgesic, anticonvulsant, anxiolytic, and neuroprotective effects in rodent models [56,57,58,59,60,61]. The detection of these enzymes in feline serum suggests the activation of biologically relevant pathways following CBD administration. Such pharmacological effects are consistent with the existing literature reporting that cannabinoids, including CBD, produce analgesic, anxiolytic, and sedative outcomes in various species [5,6,7,8,9,10,11,18]. In cats specifically, CBD has been shown to increase preoperative sedation scores and reduce intraoperative analgesic requirements [62]. Given that the MAC is influenced by agents with sedative–hypnotic and analgesic properties [63], the observed reduction in the MAC for isoflurane in this study may be attributed to these pharmacodynamic actions of CBD. Thus, the proteomic findings provide a molecular context that supports the physiological effects observed, reinforcing the hypothesis that CBD administration modulates anesthetic requirements in cats through the engagement of the endocannabinoid system.

Initially, Ultra-High-Performance Liquid Chromatography (UHPLC) was used under standard protocols to analyze serum samples. While UHPLC is a widely accepted method for detecting compounds in biological matrices, it failed to detect CBD or its metabolites in this study—likely due to serum concentrations falling below the detection limit at the 30 min sampling point. This finding aligned with previous pharmacokinetic studies in cats, which reported that serum CBD concentrations typically peak around 1–2 h after administration and exhibit considerable inter-individual variability [13,14]. Furthermore, in some fasted cats, CBD may be undetectable in serum samples collected as early as 30 min post dosing [14]. Despite this, LC-MS/MS analysis successfully identified cannabinoid biosynthetic enzymes—CBDA synthase and THCA synthase—in the sera of CBD-treated cats. The detection of these enzymes indicates that the administered product was systemically active even if CBD itself was not directly quantifiable at that early time point. Importantly, the presence of these enzymes coincided with the timeframe (15–45 min) in which increased sedation scores were observed in the preliminary phase, and aligned with the MAC-lowering effect found in our anesthetic study. This supports the hypothesis that cannabinoid-related biochemical activity was already underway within 30 min post administration, providing indirect yet physiologically meaningful evidence of early cannabinoid engagement. Therefore, the proteomic data offer a plausible mechanistic bridge between CBD exposure and anesthetic-sparing effects, even in the absence of direct CBD quantification at that time point.

However, protein annotation was based on a reference proteome database for *Cannabis sativa*, which may have introduced annotation errors or the misidentification of homologous proteins with uncharacterized functions in mammals. These findings necessitate further confirmation of the presence and biological relevance of these proteins in feline physiology. Among the 33,900 identified proteins, 12 CBD-related proteins were upregulated, with CBDA and THCA synthases showing significant upregulation. These enzymes are typically involved in cannabinoid biosynthesis in plants, suggesting that future studies utilizing targeted proteomics specific to feline samples are needed to confirm their presence and potential role in CBD metabolism. Additionally, proteins associated with redox regulation, membrane transport, and metabolic pathways were identified. While these proteins are functionally relevant to cellular processes, their specific role in feline physiology post CBD administration remains to be elucidated.

The findings of this study underscore CBD’s ability to induce distinct proteomic shifts in feline serum, which may underline its therapeutic effects. The visualization of protein clustering through heatmaps further demonstrated clear differences in protein expression before and after CBD administration. These shifts were associated with biological processes, cellular components, and molecular functions that may contribute to CBD’s anesthetic-sparing properties and broader therapeutic potential. Despite these promising findings, further research is needed to explore the impact on identified proteins and associated pathways. Comparative studies across species could provide deeper insights into CBD’s proteomic effects and its clinical applications in veterinary medicine.

Regarding method validation, the nanoscale LC-MS/MS platform was optimized for high sensitivity and specificity in detecting both small molecules and peptide fragments in feline serum. The method’s reliability was ensured through internal standardization, peak identification criteria, and controlled sample preparation workflows. However, a primary limitation of the study was the absence of protein expression validation for the cannabinoid-related enzymes detected. Although additional validation techniques, such as ELISA or Western blotting, could theoretically strengthen the reliability of our proteomic findings, the use of these methods has faced increasing scrutiny in recent years. Issues related to antibody specificity, cross-reactivity, and the limited availability of highly specific antibodies—particularly for novel or less commonly studied proteins—present significant challenges [64,65,66]. Furthermore, discrepancies in sample preparation protocols between mass-spectrometry-based proteomics and traditional immunoassays can contribute to inconsistent results. In this study, the lack of commercially available antibodies against feline CBDA synthase and THCA synthase, combined with the high cost and time required to generate custom antibodies, limited our ability to perform independent protein validation.

In particular, this study highlighted the current gaps in pharmacokinetic knowledge regarding CBD in feline models. Factors such as anesthesia, metabolic variability, and the influence of delivery formulations warrant further investigation. Future studies should aim to optimize CBD dosing strategies, including the timing and route of administration, to enhance its therapeutic efficacy. Long-term safety studies are also necessary to establish standardized protocols for clinical use, particularly in veterinary anesthesia. The findings from this study provide a foundation for understanding CBD’s molecular effects and pave the way for further research into its anesthetic-sparing properties. Advanced analytical techniques such as nanoLC-MS/MS remain indispensable for uncovering the subtle yet significant biological impacts of cannabinoids, offering new opportunities for therapeutic innovation in veterinary medicine.

## 5. Conclusions

This study supported the potential use of CBD oil as an adjunct to inhalant anesthesia in cats, demonstrating short-term safety at a 2 mg/kg dose and a reduction in anesthetic requirements. The observed decrease in the MAC suggests that CBD oil may enhance anesthesia safety by reducing inhalant concentrations, thereby minimizing dose-dependent cardiopulmonary depression. NanoLC-MS/MS effectively revealed significant protein dynamics following CBD administration. The upregulation of specific CBD-associated proteins provides a foundation for the further exploration of CBD’s role in veterinary anesthesia and treatment. Future studies should incorporate targeted proteomics and functional assays, focusing on feline-specific biological pathways, to more accurately determine the effects of CBD and validate its therapeutic potential.

## Figures and Tables

**Table 1:** Minimum alveolar concentration of isoflurane (MAC_iso_) and physiological parameters, including systolic arterial pressure (SAP), mean arterial pressure (MAP), diastolic arterial pressure (DAP), body temperature, heart rate, respiratory rate, peripheral arterial oxygen saturation (SpO_2_), and end-tidal carbon dioxide (ETCO_2_) in sixteen cats prior to (baseline) and after 2 mg/kg CBD administration. Data are presented as means ± standard deviations.

Variables	Baseline	CBD	*p*-Value
MAC_iso_	1.77 ± 0.14	1.62 ± 0.21 *	<0.001
SAP (mmHg)	100.32 ± 19.51	105.67 ± 21.31	0.095
MAP (mmHg)	76.06 ± 19.72	81.48 ± 19.59	0.081
DAP (mmHg)	61.36 ± 20.54	64.67 ± 17.51	0.201
Body temperature (°C)	38.14 ± 0.54	38.1 ± 0.50	0.770
Heart rate (beats/min)	139.66 ± 25.12	159.98 ± 27.74 *	<0.001
Respiratory rate (breaths/min)	26.66 ± 9.77	30.83 ± 12.21 *	0.005
SpO_2_	99.26 ± 1.02	99.19 ± 1.15	0.442
ETCO_2_ (mmHg)	36.51 ± 5.20	36.09 ± 4.05	0.514

* Statistically significant difference between baseline (pre-treatment) and post CBD treatment (*p* < 0.05).

**Table 2:** Complete blood count (CBC) and blood chemistry parameters in cats at baseline (before treatment) and two weeks after receiving CBD at 2 mg/kg. Variables are presented as means ± standard deviations (SDs).

Blood Parameters (Units)	Baseline	2 Weeks Post CBD	Reference Interval **	*p*-Value
Hb (g/dL)	13.9 ± 1.2	13.6 ± 1.3	9.8–15.4	0.070
Hct (%)	39 ± 3.6	38.1 ± 3.2	30–45	0.552
$\mathrm{RBC}\;({\times 10}^{6}$/µL)	9.7 ± 1.3	9.5 ± 1.5	5.0–10.0	0.623
MCV (fL)	40.9 ± 5.6	40.6 ± 5.2	39–55	0.979
MCHC (g/dL)	35.9 ± 3.1	35.6 ± 2	30–36	0.453
MCH (pg)	14.6 ± 2	14.5 ± 1.8	13–17	0.059
$\mathrm{WBC}\;({\times 10}^{3}$/µL)	9 ± 2.6	8.2 ± 2.4	5.5–19.5	0.443
$\mathrm{PLT}\;({\times 10}^{3}$/µL)	298.8 ± 125	294.5 ± 112.6	300–800	0.809
PP-Refract (g/dL)	7.4 ± 0.7	7.7 ± 0.5	6–7.5	0.138
BUN (mg/dL)	26.3 ± 6.1	22.4 ± 3.3 *	19–34	0.010
CREA (mg/dL)	1.5 ± 0.2	1.5 ± 0.2	0.9–2.2	0.796
ALT (U/L)	48.8 ± 16.7	54.8 ± 29.7	25–97	0.836
AST (U/L)	23.7 ± 6.3	28.1 ± 8.6	7–38	0.178
ALP (U/L)	36.8 ± 13.7	35.4 ± 14	0–45	0.208
TP (g/dL)	7.4 ± 0.6	7.4 ± 0.5	6.0–7.9	0.390
ALB (g/dL)	3.6 ± 0.3	3.5 ± 0.3	2.8–3.9	0.103

Hb = hemoglobin, Hct = Hematocrit, RBC = red blood cell count, MCV = mean corpuscular volume, MCHC = mean corpuscular hemoglobin concentration, MCH = mean corpuscular hemoglobin, WBC = white blood cell count, PLT = platelet count, PP-Refract = plasma protein measured using a refractometer, BUN = Blood urea nitrogen, CREA = Creatinine, ALT = alanine aminotransferase, AST = aspartate aminotransferase, ALP = alkaline phosphatase, TP = total protein, and ALB = Albumin. * Significant difference (*p* < 0.05). ** Reference intervals were determined based on veterinary laboratory guidelines [33].

**Table 3:** Nominated proteins based on biological process, cellular components, and molecular functions involvement using UniProtKB/Swiss-Prot.

Protein ID	Protein Names	Gene Names	Fold Change (FC)	Log2(FC)	Biological Process	Cellular Component	Molecular Function
A0A1V0QSG9	Terpene synthase	N/A	1.5295	0.61307	diterpenoid biosynthetic process [GO:0016102]	N/A	magnesium ion binding [GO:0000287]; terpene synthase activity [GO:0010333]
A0A1V0QSG6	GPPS small subunit	N/A	1.4085	0.49414	diterpenoid biosynthetic process [GO:0016102]	N/A	magnesium ion binding [GO:0000287]; terpene synthase activity [GO:0010333]
A0A3G5EA60	Tetrahydrocannabinolic acid synthase	N/A	1.1744	0.23194	N/A	N/A	FAD binding [GO:0071949]; oxidoreductase activity [GO:0016491]
A0A1V0QSF9	Terpene synthase	N/A	1.1415	0.19093	diterpenoid biosynthetic process [GO:0016102]	magnesium ion binding [GO:0000287]; terpene synthase activity [GO:0010333]	diterpenoid biosynthetic process [GO:0016102]
A0A1V0QSF4	Terpene synthase	N/A	1.1366	0.18475	diterpenoid biosynthetic process [GO:0016102]	magnesium ion binding [GO:0000287]; terpene synthase activity [GO:0010333]	diterpenoid biosynthetic process [GO:0016102]
A0A1V0QSF6	Terpene synthase	N/A	1.0844	0.11687	diterpenoid biosynthetic process [GO:0016102]	magnesium ion binding [GO:0000287]; terpene synthase activity [GO:0010333]	diterpenoid biosynthetic process [GO:0016102]
A0A0E3TIL5	Cannabidiolic acid synthase	N/A	1.0833	0.11548	N/A	N/A	FAD binding [GO:0071949]; oxidoreductase activity [GO:0016491]
A0A3G5EBM5	Cannabidiolic acid synthase	N/A	1.0833	0.11548	N/A	N/A	FAD binding [GO:0071949]; oxidoreductase activity [GO:0016491]
A0A7J6FVY1	Expansin-like CBD domain-containing protein	F8388_020318	1.0833	0.11548	N/A	N/A	N/A
A0A7C9FBI5	Tetrahydrocannabinolic acid synthase	THCAS	1.0833	0.11548	N/A	membrane [GO:0016020]	FAD binding [GO:0071949]; oxidoreductase activity [GO:0016491]
A0A7C9B3L6	Cannabidiolic acid synthase	CBDAS	1.0695	0.096959	N/A	N/A	FAD binding [GO:0071949]; oxidoreductase activity [GO:0016491]
F1LKH6	Polyketide synthase 1	PKSG1	1.0362	0.051353	polyketide biosynthetic process [GO:0030639]	cytoplasm [GO:0005737]	acyltransferase activity, transferring groups other than amino-acyl groups [GO:0016747]

## Data Availability

The data presented in this study have been included within the article. The raw data supporting the findings of this study are available on request from the corresponding author. The MS/MS raw data and analysis files have been deposited with the ProteomeXchange Consortium (http://proteomecentral.proteomexchange.org, accessed on 24 March 2025) via the jPOST partner repository (https://jpostdb.org, accessed on 8 May 2025) with the data set identifiers JPST003725 and PXD062262 (preview URL for reviewers: https://repository.jpostdb.org/preview/91390514767e40d5bde386, accessed on 8 May 2025. Access key: 8710).

## Supplementary Materials

The following supporting information can be downloaded at: https://www.mdpi.com/article/10.3390/ani15101393/s1, Table S1: A total of 46 CBD-associated proteins of *Cannabis sativa* (hemp/marijuana), based on reviewed entries in the UniProt Swiss-Prot database (as of 1 January 2025); Table S2: The list of 33900 proteins from *Cannabis sativa* (hemp)(marijuana) that were matched in all serum samples using in solution digestion coupled with LC-MS/MS; Table S3: The list of 30 proteins highlights CBD-induced changes in feline expression patterns (paired fold change 17 proteins, along with 13 THCA/CBDA synthase proteins before and after CBD administration).

## Abbreviations

The following abbreviations have been used in this manuscript:

CBD	Cannabidiol
OA	Osteoarthritis
MAC	Minimum alveolar concentration
MAC_iso_	Minimum alveolar concentration of isoflurane
MAC_baseline_	Baseline minimum alveolar concentration of isoflurane
MAC_CBD_	Minimum alveolar concentration of isoflurane after CBD administration
ECS	Endocannabinoid system
THC	Tetrahydrocannabinol
BUN	Blood urea nitrogen
ALT	Alanine aminotransferase
CBDA	Cannabidiolic acid
CBG	Cannabigerol
DSH	Domestic shorthair
SpO_2_	Peripheral arterial oxygen saturation
ETCO_2_	End-tidal carbon dioxide partial pressure
HR	Heart rate
RR	Respiratory rate
ECG	Electrocardiography
SAP	Systolic arterial blood pressure
MAP	Mean arterial blood pressure
DAP	Diastolic arterial blood pressure
CO_2_	Carbon dioxide
N_2_O	Nitrous oxide
SD	Standard deviation
IQR	Interquartile range
NH_4_HCO_3_	Ammonium bicarbonate
FA	Formic acid
nanoLC-MS/MS	Nanoscale liquid chromatography–tandem mass spectrometry
CAD	Collision-activated dissociation
DDA	Data-dependent acquisition
MS1	Single-stage mass spectrometry
MS2	Tandem mass spectrometry
DP	Declustering potential
CE	Capillary electrophoresis
FDR	False discovery rate
THCA	Tetrahydrocannabinolic acid
Hb	Hemoglobin
Hct	Hematocrit
RBC	Red blood cell count
MCV	Mean corpuscular volume
MCHC	Mean corpuscular hemoglobin concentration
MCH	Mean corpuscular hemoglobin
WBC	White blood cell count
PLT	Platelet count (number of platelets in the blood)
PP-Refract	Plasma protein measured using a refractometer
CREA	Creatinine
AST	Aspartate aminotransferase
ALP	Alkaline phosphatase
TP	Total protein
ALB	Albumin
THCAS	Tetrahydrocannabinolic acid synthase
CBDAS	Cannabidiolic acid synthase
FC	Fold Change
CB1	Cannabinoid receptor type 1
GABAA	Gamma-aminobutyric acid type A
FAAH	Fatty acid amide hydrolase
AEA	Anandamide
CYP	Cytochrome P450
UHPLC	Ultra High-Performance Liquid Chromatography

## Appendix A

**Table A1:** Sedation scoring system using a numerical rating scale adapted from previously established sedation assessment methods in cats [19,20,21,22].

Criteria	Score
Completely awake, able to stand and walk, normal posture	0
Stands but staggers when attempting to walk	1
Sternal recumbency, able to lift head up, unable to stand, may makes weak attempts to rise	2
Lateral recumbency, responds to gentle stroking, a moving toy, or handclapping (able to slightly lift the head, tail, or a limb)	3
Lateral recumbency, does not respond to gentle stroking, a moving toy, or handclapping	4
